# Parental Depression and Anxiety Associated with Newborn Bloodspot Screening for Rare and Variable-Onset Disorders

**DOI:** 10.3390/ijns8040059

**Published:** 2022-11-10

**Authors:** Natalie A. Boychuk, Niamh S. Mulrooney, Nicole R. Kelly, Aaron J. Goldenberg, Ellen J. Silver, Melissa P. Wasserstein

**Affiliations:** 1 Department of Pediatrics, Albert Einstein College of Medicine and Children’s Hospital at Montefiore, Bronx, NY 10467, USA; 2 Department of Bioethics, Case Western Reserve University School of Medicine, Cleveland, OH 44106, USA

**Keywords:** newborn screening, ELSI, psychological

## Abstract

The ability to screen newborns for a larger number of disorders, including many with variable phenotypes, is prompting debate regarding the psychosocial impact of expanded newborn bloodspot screening (NBS) on parents. This study compares psychological outcomes of parents of children with a range of NBS/diagnostic experiences, with a particular focus on lysosomal storage disorders (LSDs) and X-linked adrenoleukodystrophy (X-ALD) as representative disorders with complex presentations. An online cross-sectional survey with six domains was completed in 2019 by a volunteer sample of parents with at least one child born between 2013 and 2018. Parents were classified in the analysis stage into four groups based on their child’s rare disorder and means of diagnosis. Stress and depression were estimated using dichotomous measures of the depression subscale of the Hospital Anxiety and Depression Scale and the Parental Stress Scale. Logistic regression models were estimated for the relationship between the parent group and stress/depression, controlling for demographic variables (region of the US, income, education, major life events, relationship to the child, number of children, parent age, and race/ethnicity). One hundred seventy-four parents were included in this analysis. Parents of children with an LSD or X-ALD diagnosis clinically may have higher odds of depression (OR: 6.06, 95% CI: 1.64–24.96) compared to parents of children with the same disorders identified through NBS, controlling for covariates. Although a similar pattern was observed for parental stress (OR: 2.85, 95% CI: 0.82–10.37), this did not reach statistical significance. Ethically expanding NBS and genome sequencing require an understanding of the impacts of early detection for complex disorders on families. These initial findings are reassuring, and may have implications as NBS expands. Given our small sample size, it is difficult to generalize these findings to all families. These preliminary trends warrant further investigation in larger and more diverse populations.

## 1. Introduction

Since the development of the newborn bloodspot screening (NBS) test for phenylketonuria (PKU) in 1963 [1], NBS technology has advanced substantially with the invention of multiplex biochemical assays and DNA-based screening. This has enabled NBS programs to screen for more disorders, with most states now screening for at least 30 disorders on their routine panel [2]. Some current NBS disorders, and many others under consideration for inclusion on NBS panels, such as lysosomal storage disorders (LSDs) and X-linked Adrenoleukodystrophy (X-ALD), have complex clinical manifestations. LSDs encompass more than 50 disorders, and these, combined, occur in between 1:1500 and 1:7000 live births [3]. For most LSDs, there is a broad phenotypic range, and the later-onset forms may be more prevalent than early-onset forms for some LSDs [4,5]. X-ALD is a metabolic disorder that occurs in about 1:17,000 live births [6]. Like LSDs, X-ALD has a broad spectrum of clinical severity, ranging from childhood-onset neurodegenerative disease to adult-onset disease with primarily adrenal manifestations [7].

Most current NBS methods for LSDs, X-ALD, and other disorders are unable to determine whether abnormal results are predictive of early- or late-onset phenotypes [5]. Accordingly, individuals may be diagnosed shortly after birth with disorders that may not present until adulthood, raising ethical questions and challenging the traditional purpose of newborn screening: to identify individuals at risk for serious disorders that manifest in infancy or childhood [8]. Some empirical studies suggest that NBS for later-onset disorders may inflict harm on families, who must live with uncertainty around if, or when, their child may begin experiencing symptoms, thus potentially turning families into “patients in waiting [9].” For example, interviews with parents of children diagnosed with X-ALD through NBS revealed stress and difficulty living with uncertainty [10]. However, recent discussions have challenged some of these assumptions, especially when the harm to newborns and their families is not fully understood. These arguments focus on the ability of families to cope with complex genetic information and reflect the potential value that later-onset information may have for promoting the best long-term interests of a child [11].

Thus, it is critical to obtain empiric data about the existence, severity, and endurance of the potential harm of NBS for complex disorders, particularly as we approach genome-based NBS. To this end, we describe the psychological outcomes of parents whose children were diagnosed via NBS with disorders with variable ages of onset (LSDs, X-ALD), as compared to parents with other experiences with NBS and rare disorders. Our goal was to assess the impact of early detection on families, as well as to inform screening policies and practices by increasing our understanding of the potential benefits and harms of expanding NBS panels. This study was conducted within the context of a NBS pilot study that was conducted in five ethnically diverse hospitals with high birth rates in New York, which screened over 65,000 newborns for five lysosomal storage disorders (LSDs) [12].

## 2. Materials and Methods

### 2.1. Ethics

This study was approved by the Institutional Review Board at the Albert Einstein College of Medicine (IRB Number: 2016-7183). Informed consent was obtained via an information page at the opening of the survey.

### 2.2. Eligibility

English- or Spanish-speaking parents with at least one child born in the United States (US) between 2013 and 2018 were eligible to participate.

### 2.3. Recruitment and Survey Procedure

This study aimed to recruit parents of children with a range of NBS and/or rare disease experiences. In order to obtain a sufficient number of responses from parents of children with the rare outcome of a positive Recommended Uniform Screening Panel (RUSP) or LSD/X-ALD NBS result, a targeted convenience sample of parents was recruited. A recruitment target of at least 97 participants in total was set prior to study implementation in order to detect group differences of moderate effect sizes, with 80% power and 5% significance, based on basic sample size calculation results. Our approach to recruitment was tiered and multi-faceted: families with an infant identified through the pilot NBS study for LSDs in New York, who were clinically followed by study investigators, were invited to participate through study letters written by the physicians. Additionally, study information was sent to select NBS website administrators (e.g., state-specific newborn screening websites), patient advocacy groups (PAGs), and disease-specific websites and Facebook groups, with the request to share study details along with a link to participate through their social media channels. Study posters were also posted in select pediatric clinics affiliated with the Children’s Hospital at Montefiore (CHAM) (Bronx, NY). Pediatricians and other pediatric providers at CHAM received basic information about the study through Grand Rounds presentations and email notifications [12]. Lastly, study materials were sent to providers at inherited metabolic referral centers in seven states that were screening for at least one LSD and/or X-ALD between 2013 and 2018. They were asked to share study information with patients during clinical encounters. 

This survey launched in June 2019 and was open for three months. This ensured that any parent of a child with a rare condition was at least six months post-diagnosis, as the literature on the parent burden among children with cancer has suggested that the psychological burden may be heightened in parents immediately following their child’s diagnosis, and this pattern may hold across other conditions [13]. Parents who learned of the survey through any of these outreach channels followed the link to voluntarily complete the survey through Qualtrics, a secure online survey development software. The survey was expected to take 20–30 min to complete and was available in English and Spanish. Parents who completed the survey received a $30 gift card.

### 2.4. Measures

A cross-sectional survey with up to 70 questions spanning six domains (demographics, attitudes towards NBS, child’s NBS results, PSS, HADS-D, and health profile of children born before 2013 with a diagnosis of an LSD/X-ALD) was developed. 

Parents were classified into groups in the analysis stage based on their response to questions regarding their child’s NBS result and/or rare disease diagnosis. Parents were grouped into the following categories if they had at least one child: (1) diagnosed with an LSD or X-ALD through NBS (LSD-NBS Dx), (2) diagnosed with an LSD or X-ALD because of clinical presentation or family history (LSD-Other Dx), and (3) diagnosed with a routine/RUSP disorder through NBS (RUSP-NBS Dx). The LSDs included Fabry Disease, Gaucher Disease, Mucopolysaccharidosis type I (MPS I), Pompe Disease, Krabbe Disease, and Acid Sphingomyelinase Deficiency (ASMD or Niemann Pick A, A/B, and B). Disorders were classified as “routine” based on the 2016 RUSP. Parents were included in the LSD or RUSP groups if they had at least one child diagnosed with any disorder in question, even if they had other healthy children. The fourth group, who were also a convenience sample recruited using the strategies described above, served as a comparison and consisted of parents of children with no rare disease diagnoses (No Dx). This group was based on parent reports of their child’s normal NBS results and no rare disease diagnoses. 

We used the depression subscale of the Hospital Anxiety and Depression Scale (HADS) [14] and the Parental Stress Scale (PSS) [15] to assess psychological wellbeing in parents. The HADS depression subscale has seven scored items. Scores range from 0 to 21, and a score ≥ 8 indicates symptoms of depression [14,16]. This scale was selected because it has been used and validated in previous research characterizing parental anxiety and depression, including research assessing anxiety and depression in parents of children with cystic fibrosis [17], children with cancer [18], and very preterm infants [19]. While the HADS includes both anxiety and depression, only the depression subscale (HADS-D) was assessed in this research, as anxiety was not an outcome of interest. The PSS contains 18 items measuring perceived stresses of parenthood. Scores range from 18 to 90, with a higher score representing a higher level of parenting stress [20]. The PSS was selected because it has been validated by parents of children with a range of health experiences and across cultures [21]; additionally, the scale uniquely captures both positive and negative attributes of parental stress and fulfillment. Generally, scores among parents of healthy children or with children of unknown health status have been found to range from 37.1 to 38.81 [15,22]. Race and ethnicity were collected in a single self-report measure, and participants selected all identities that applied to them from a list of racial identities (e.g., Asian, Black or African American, Middle Eastern or Arab American, White, Hispanic or Latino/a/x ethnicity). Participants could opt out of reporting race and ethnicity. Due to small sample sizes in each category, Hispanic or Latino/a/x ethnicity was assessed as a separate binary variable (Hispanic or Latino/a/x identity or no) and race was aggregated into three categories: White (non-Hispanic or Latino/a/x), Black or African American (non-Hispanic or Latino/a/x), and all other races, which included individuals of multiple racial identities and/or individuals who identified as Hispanic or Latino/a/x. In the context of this study, race is understood as a social construct and was included in our analysis in order to surface disparities in access to care rooted in systemic racism [23].

Education, relationship status, number of children born between 2013 and 2018, state, income, and employment status were also aggregated due to sample size concerns. The state of residence variable was aggregated to region of the US using the Census Bureau Regions and Divisions [24]. A dichotomous question about major life changes (i.e., “In the past year, have you experienced any major life changes that have caused you stress?”) was captured as a potential confounding variable to control for the impact of external factors (e.g., death in the family, loss of employment, etc.) on psychological health.

### 2.5. Statistical Analysis 

Data were analyzed using R version 4.1.0. HADS and PSS were transformed into binary measures due to observed bimodal distributions of both outcomes. A HADS score ≥ 8 was defined as “Considerable Depression” and <8 was defined as “Not Considerable Depression.” No validated cut-off for severe parental stress was identified; therefore, we used a median split to binarize PSS scores. All scores < 41 were classified as “low parental stress” and >41 as “high parental stress.” Given that the peaks in the distribution were observed to be substantially above and below the median, this approach was considered appropriate. 

Descriptive statistics were computed in order to characterize the sociodemographic distribution of the study sample. Explanatory analyses were conducted in order to explore the association between parent group and parental stress and depression. If a participant responded, “Do Not Wish to Answer,” they were excluded when analyzing that item. 

Chi-square tests or Fisher’s Exact tests were used to examine bivariate relationships of the parent group and sociodemographic variables with depression and stress outcomes. Two-sample t-tests were used to assess the correlations between parent age and depression and stress. Cramer’s V and Cohen’s D were computed as effect size measures. 

Binary logistic regression models were constructed in order to assess the relationships between diagnostic group and the outcomes of considerable depression or high parental stress score. Sociodemographic variables that were significantly associated with depression/stress score at the 10% level of significance in binary analyses were included in the models. Generalized VIF values were computed in order to assess collinearity, and a score of greater than 2.5 was considered substantial collinearity.

## 3. Results

### 3.1. Description of the Study Sample

In total, 174 parents were included in this analysis with the following group distribution: LSD-NBS Dx (n = 20), LSD-Other Dx (n = 41), RUSP-NBS Dx (n = 52), and No Dx (n = 61). A distribution of disorders in children as self-reported by their parents is included as a supplementary material (Supplementary Table S1). Participants’ ages ranged from 20 to 43, with an average age of 30 (SD: 3.9). Most parents who responded were married or living with a partner (93%), had received a bachelor’s degree or higher (48%), were employed full- or part-time (84%), and identified themselves as the mother of the child (63%). More than half (57%) of participants reported annual household income of more than $50,000, and the majority of participants self-identified as White (58%) and not Hispanic or Latino/a/x (91%) (Table 1).

### 3.2. Levels of Depression

**Figure 1:**
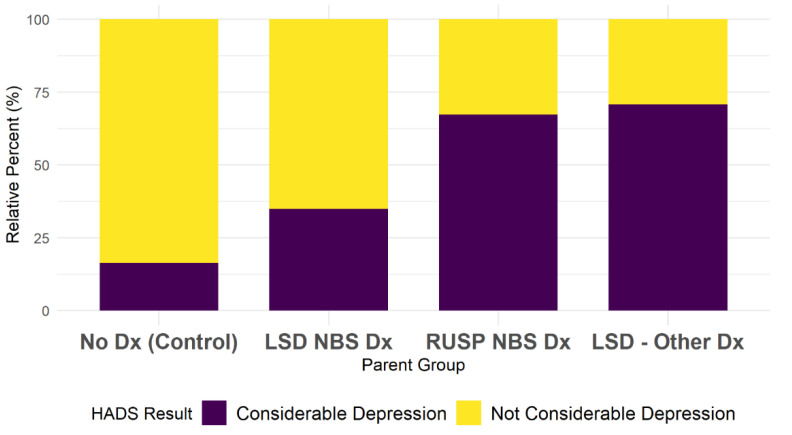
Depression outcome by parent group.

There was a significant relationship between parent group and depression (*p* < 0.001), with a relatively strong (Cramer’s V = 0.49) effect size. A higher proportion of participants in the LSD-Other Dx (71%) and RUSP-NBS Dx (67%) groups had a considerable depression score as compared to parents in the LSD-NBS Dx (35%) and No Dx (16%) groups (Figure 1). 

Several sociodemographic variables were found to be significantly associated with depression in bivariate analyses with small to moderate effect sizes, including income level (*p* = 0.003, Cramer’s V = 0.29), race (*p* = 0.05, Cramer’s V = 0.18), and region of the US (*p* = 0.001, Cramer’s V = 0.30) (Table 2).

A binary logistic regression model found that LSD-Other Dx parents had higher odds of having a considerable depression score than LSD-NBS Dx parents, holding their region of the US, race, and income constant (OR = 6.06, 95% CI: 1.64–24.96) (Table 3). Similarly, RUSP-NBS Dx parents had higher odds of receiving a considerable depression score compared with parents in the LSD-NBS Dx group (OR = 3.44, 95% CI: 1.00–12.88), when holding their region, race, and income constant. Generalized VIF values ranged from 1.03 to 1.25, suggesting that there was no substantial collinearity between predictors.

### 3.3. Levels of Parental Stress 

**Figure 2:**
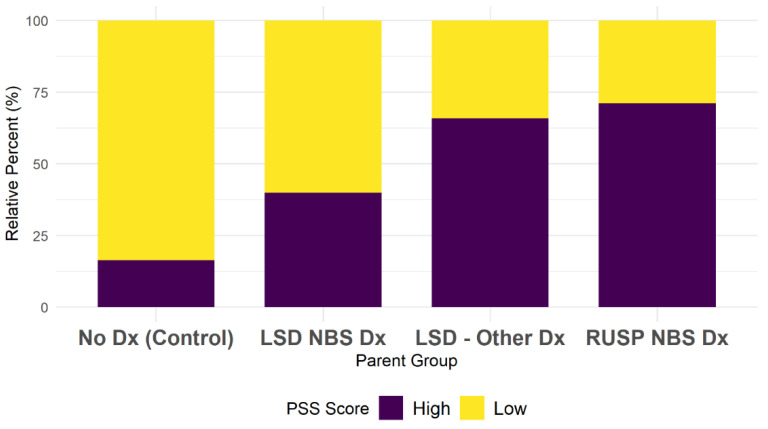
Parental stress scale score outcome by parent group.

Chi-square tests revealed significant differences between the diagnostic groups and the binary parental stress outcomes (*p* < 0.001, Cramer’s V = 0.49). Higher proportions of parents in the LSD-Other Dx (66%) and RUSP-NBS Dx (71%) groups had high parental stress scores as compared to parents in the LSD-NBS Dx (40%) and No Dx (16%) groups (Figure 2). 

Income (*p* = 0.03, Cramer’s V = 0.24), employment status (*p* = 0.006, Cramer’s V = 0.22), region of the US (*p* < 0.001, Cramer’s V = 0.32), relationship to the child (*p* = 0.07, Cramer’s V = 0.14), and reportedly experiencing at least one major life event in the last year (*p* = 0.02, Cramer’s V = 0.18) were significantly associated with parental stress with weak to moderate strength, and were included in the logistic regression model (Table 2). 

A binary logistic regression model assessing the relationship between parental stress and parent group found a pattern in which parents in the LSD-Other Dx group had, on average, 2.85 times the odds of having a high parental stress score (95% CI: 0.82–10.37) as compared to parents in the LSD-NBS Dx group, holding their relationship to the child, major life changes, employment status, region, and income level constant (Table 3). Similarly, a tendency was observed in which parents in the RUSP-NBS Dx group had greater odds of having a high parental stress score (OR = 3.20, 95% CI: 0.96–11.10) compared to LSD-NBS Dx parents, controlling for covariates, although this did not reach significance. Generalized VIF values were less than 1.37, indicating no substantial multicollinearity in the model.

## 4. Discussion

NBS for disorders with later-onset phenotypes poses ethical challenges that should be addressed in order to appropriately implement expanded screening. A central concern lies in extending the possible implications of NBS into later childhood, adolescence, and adulthood, as well as what longer-term psychosocial impact this may have on families and pediatric practice. Additionally, some argue that the “unbearable certainty of knowing” causes significant harm to families of children with potentially later-onset conditions who receive an early diagnosis through NBS [5]. The American Society of Human Genetics, the American Academy of Pediatrics, and other professional societies have historically recommended against screening infants for later-onset disorders, suggesting that testing be deferred until adulthood unless a treatment administered in childhood could reduce morbidity and mortality [25,26].

Conditions with variable ages of onset create a complicated scenario in the context of NBS, given that some phenotypes lead to an onset that falls within an accepted timeframe for early disclosure, while others may lead to later or even adult-onset forms, for which we may wish to protect a child’s right to know, or not know, that information when they reach a stage of life with higher decisional capacity. 

Despite these concerns, limited data suggest that the harm may be less significant than previously anticipated. A study of the psychological impact of receiving expanded NBS results found no significant differences in anxiety, stress, or depression among mothers of newborns who received true negative, true positive, or false positive NBS results. The study used the Parental Stress Index (PSI), an original source of the PSS scale, to assess the impact of NBS on maternal well-being [27]. Similarly, a study of stress among parents of children with a genetic disorder found that mothers of children identified through NBS had significantly lower PSI scores than mothers whose children were identified clinically [28]. This may be partially explained by improved health outcomes in those children diagnosed through NBS, who required lower rates of hospitalization and lower rates of diagnosis of intellectual disability, compared with children diagnosed clinically [28].

The findings of this study affirm previous data regarding the impact of NBS on psychosocial wellbeing. Our study suggests that parents who have a child with an LSD/X-ALD diagnosed via NBS have lower odds of experiencing depression or parental stress than parents of children diagnosed with these same disorders clinically. While a rare disorder is almost never welcome, this suggests that parents may experience a relatively healthier psychological state than they otherwise would have, had their child received the diagnosis after the onset of symptoms. 

There are several potential reasons for this. First, NBS may reduce certain elements of the diagnostic odyssey for families. There is evidence regarding the psychological toll that this often long, expensive, and anxiety-ridden search has on parents [29]. Even for later-onset variants, early detection may provide parents with more certainty about their newborn’s condition while they seek information and follow-up services. Second, NBS allows parents to be active participants in their child’s care at the earliest possible stages of disease, offering parents more control than if the child were diagnosed later. There is a direct correlation between perceived control over one’s situation and feelings of anxiety and depression, so it is possible that NBS may improve psychosocial outcomes by giving parents a greater degree of control [30,31]. 

Interestingly, a pattern was observed in which parents in the LSD-NBS Dx group had lower odds of experiencing depression or stress than parents in the RUSP-NBS Dx group. It is unclear why this tendency exists, but it may be due in part to the nature of the LSDs/X-ALD as compared to other NBS disorders (e.g., slower vs. more rapid onset, non-life-threatening vs. life-threatening) and treatment options (e.g., bi-weekly infusions vs. permanent dietary restrictions). Additionally, the data on our LSD-NBS Dx group may have been skewed by parents who participated in our pilot NBS program [12], which included parent education, consent, and clear, timely communication of results. One study suggests that a family-centered genetic counselling intervention for parents of children who have received abnormal NBS results for cystic fibrosis, which included addressing parental information needs, could decrease parental distress following their child’s diagnosis [32]. It is unclear whether NBS pilot studies that provide parents basic information during the consenting process may similarly influence parental emotions associated with NBS. Because of this, some LSD-NBS Dx parents may have been somewhat prepared for the possible outcome of a positive screen, which may have resulted in reduced stress and anxiety compared to parents who did not receive similar pre-NBS education. Additional research regarding pre-NBS education and parental psychological wellbeing is needed to better understand this potential relationship.

The study has several limitations. First, our small overall sample size limited statistical power. This is reflected in the wide confidence intervals observed in all odds ratios. Almost all sociodemographic categories were aggregated in order to minimize this limitation, which reduced the granularity of our findings. Understanding that sociodemographic aggregation can mask important differences in experiences with the health system, this aggregation presents a significant limitation to the study. In particular, Hispanic or Latino/a/x ethnicity was assessed in bivariate analysis but failed to reach significance, potentially due to small sample sizes, and racial identity was trichotomized in order to manage sample size concerns. 

The study group was recruited mainly through PAGs and the medical community, and, therefore, may have been self-selective. Some research has shown that parents who join PAGs tend to be White, have an annual income around the national average, live in a suburban area, be college graduates, and be either married or living with a partner; this is consistent with the demographic breakdown of this study [33]. Future studies should recruit larger and more diverse samples in order to surface disparities in impacts of NBS and to increase the generalizability of our findings. Additionally, high levels of parental stress and depression were observed in the RUSP-NBS Dx group. This interesting finding may reflect the different stressors associated with the nature of the diseases and treatment obligations, but it may also suggest the presence of selection bias. Parents who were recruited through metabolic clinics and patient support groups may be more likely to have a child with a more severe form of a RUSP disorder than the general population of parents of children with RUSP disorders, who were identified through NBS. 

We did not collect baseline parental stress and depression scores or ask participants if they had any previous or current psychological disorders. Additionally, the time elapsed between receipt of results and completion of the survey was not consistent across participants. Some research suggests that emotions are more intense immediately after a diagnosis [34]. We attempted to mitigate this effect by releasing the survey in order to maximize the participants responding to the survey at least six months after their child’s diagnosis; however, further restriction based on the time since diagnosis was not possible because of the small sample size. 

We did not collect information about how parents received NBS results (e.g., pilot study, supplementary screening, routine screening), making it difficult to reach conclusions about the impact of the mode of NBS on parental outcome. Another limitation was that we did not have a sufficient sample size to analyze the LSD-NBS Dx and LSD-Other Dx groups by anticipated age of onset or disease severity. Larger studies will be required to enable this level of granularity.

Finally, of the two HADS subscales, we only used the depression subscale. One study suggested that HADS is more appropriate as a summed measure of general psychological distress, rather than a tool for specific symptoms of depression or anxiety [35]. However, anxiety was not an outcome of interest for this study, so the HADS-D was used in conjunction with the PSS.

## 5. Conclusions

This study suggests that parents who receive their child’s diagnosis for one of these disorders through NBS have lower odds of depression compared with parents of children diagnosed with these disorders through family history or clinical presentation. A similar pattern emerged regarding parental stress, although this finding failed to reach significance. These findings may challenge traditional concerns regarding the psychosocial harms of expanded NBS, and they also suggest that the benefits of early identification may outweigh the risks for many families. Additionally, these findings add to our understanding of the impact that screening can have on families, which in turn can help NBS programs and healthcare providers provide better support for newborns and families with positive results. As there is continued debate about the risks and benefits of expanded newborn screening, additional research will be needed to understand the psychological implications of false-positive results for families, who may have substantially different experiences and needs than parents of children with true positive results. Future research should also build on these results by assessing larger, more diverse sample populations, and should qualitatively analyze parental experiences in order to further explain these observed patterns. 

## Figures and Tables

**Table 1:** Sociodemographic characteristics of parents who participated in the survey.

	All N = 174	LSD-NBS Dx n = 20	LSD-Other Dx n = 41	RUSP-NBS Dx n = 52	No Dx n = 61
Age, years (mean ± SD)	30.00 ± 3.92	30.10 ± 3.57	30.34 ± 3.83	28.87 ± 3.55	30.70 ± 4.24
Relationship to child					
Mother	110 (63%)	12 (60%)	25 (61%)	32 (62%)	41 (67%)
Father	64 (37%)	8 (40%)	16 (39%)	20 (38%)	20 (33%)
Relationship Status					
Married or living with a partner	162 (93%)	18 (90%)	41 (100%)	48 (92%)	55 (90%)
Not married or living with a partner	12 (7%)	2 (10%)	0 (0%)	4 (8%)	6 (10%)
N Children Born Between 2013–2018					
One	113 (65%)	16 (80%)	25 (61%)	35 (67%)	37 (61%)
More than one	61 (35%)	4 (20%)	16 (39%)	17 (33%)	24 (39%)
Education					
Bachelor’s degree or higher ^a^	84 (48%)	11 (55%)	22 (54%)	24 (46%)	27 (44%)
Less than a bachelor’s degree ^b^	89 (51%)	9 (45%)	18 (44%)	28 (54%)	34 (56%)
Do not wish to answer	1 (1%)	0 (0%)	1 (2%)	0 (0%)	0 (0%)
Employment					
Employed full-time or part-time	147 (84%)	16 (80%)	37 (90%)	47 (90%)	47 (77%)
Stay at home parent	22 (13%)	3 (15%)	3 (7%)	4 (8%)	12 (20%)
Unemployed or unable to work	5 (3%)	1 (5%)	1 (2%)	1 (2%)	2 (5%)
Income (annual household)					
Less than $34,999	22 (13%)	2 (10%)	2 (5%)	5 (10%)	13 (21%)
$35,000–$49,999	42 (24%)	2 (10%)	13 (32%)	15 (29%)	12 (20%)
$50,000–$99,999	81 (47%)	12 (60%)	22 (54%)	27 (52%)	20 (33%)
More than $100,000	18 (10%)	2 (10%)	3 (7%)	4 (8%)	9 (15%)
Do not wish to answer	11 (6%)	2 (10%)	1 (2%)	1 (2%)	7 (12%)
US Region					
Midwest	32 (18%)	2 (10%)	8 (20%)	6 (12%)	16 (26%)
Northeast	43 (25%)	7 (35%)	7 (17%)	7 (13%)	22 (36%)
South	40 (23%)	7 (35%)	12 (29%)	14 (27%)	7 (11%)
West	58 (33%)	4 (20%)	14 (34%)	25 (48%)	16 (26%)
Race					
White, non-Hispanic/Latino/a/x	100 (57%)	12 (60%)	30 (73%)	26 (50%)	32 (52%)
Black/African American, non-Hispanic/Latino/a/x	28 (16%)	3 (15%)	6 (15%)	5 (10%)	14 (23%)
All other races/multiple races, and/or Hispanic/Latino/a/x	45 (26%)	5 (25%)	5 (12%)	21 (40%)	14 (23%)
Do not wish to answer	1 (1%)	0 (0%)	0 (0%)	0 (0%)	1 (2%)
Ethnicity					
Hispanic or Latino/a/x	14 (8%)	2 (10%)	1 (2%)	6 (12%)	5 (8%)
Not Hispanic or Latino/a/x	159 (91%)	18 (90%)	40 (98%)	46 (88%)	55 (90%)
Do not wish to answer	1 (1%)	0 (0%)	0 (0%)	0 (0%)	1 (2%)

^a^ Bachelor’s degree, master’s degree, advanced graduate work (e.g., JD, MD, PhD, etc.). ^b^ Less than a high school diploma, high school diploma or equivalent (i.e., GED), some college but no degree, associate degree, or vocational, technical, or other types of training.

**Table 2:** Results of bivariate analyses for the association between covariates and HADS/PSS outcomes.

HADS					
	Chi-Square Statistic or Fisher’s Exact Test	*p*-Value	Cramer’s V		
Region of the US	$X^{2}=$ 15.63	*p* = 0.001 ***	V = 0.30		
Income	$X^{2}=$ 13.68	*p* = 0.003 ***	V = 0.29		
Race	$X^{2}=$ 5.82	*p* = 0.05 *	V = 0.18		
Relationship to child	$X^{2}=$ 2.69	*p* = 0.10	V = 0.12		
Number of children	$X^{2}=$ 2.66	*p* = 0.10	V = 0.12		
Employment status	FET	*p* = 0.15	V = 0.15		
Education	$X^{2}=$ 0.75	*p* = 0.39	V = 0.07		
Ethnicity (Hispanic or Latino/a/x, y/n)	$X^{2}=0.65$	*p* = 0.42	V = 0.06		
Major life events (y/n)	$X^{2}=$ 0.44	*p* = 0.51	V = 0.05		
Relationship status	$X^{2}=0.06$	*p* = 0.80	V = 0.02		
	n	Mean	T-value (95% CI)	*p*-value	Cohen’s D
Not considerable depression	93	30.33	−1.22 (−1.87–0.44)	0.22	−0.18
Considerable depression	81	29.62			
**PSS**					
	**Chi-Square Statistic or Fisher’s Exact Test**	***p*** **-Value**	**Cramer’s V**		
Region of the US	$X^{2}=$ 17.41	*p* < 0.001 ***	V = 0.32		
Employment	FET	*p* = 0.006 ***	V = 0.22		
Major life events (y/n)	$X^{2}=$ 5.76	*p* = 0.02 **	V = 0.18		
Income	$X^{2}=$ 9.21	*p* = 0.03 **	V = 0.24		
Relationship to the child	$X^{2}=$ 3.38	*p* = 0.07 *	V = 0.14		
Relationship status	$X^{2}=$ 1.98	*p* = 0.16	V = 0.11		
Race	$X^{2}=$ 2.91	*p* = 0.23	V = 0.13		
Education	$X^{2}=$ 0.74	*p* = 0.48	V = 0.07		
Number of children	$X^{2}=$ 0.21	*p* = 0.65	V = 0.04		
Ethnicity (Hispanic or Latino/a/x y/n)	$X^{2}=0.04$	*p* = 0.84	V = 0.02		
	n	Mean	T-Value (95% CI)	*p*-Value	Cohen’s D
Low PSS	92	30.32	−1.13 (−1.84–0.50)	0.26	−0.17
High PSS	82	29.65			

* Significant at *p* < 0.10. ** Significant at *p* < 0.05. *** Significant at *p* < 0.01.

**Table 3:** Exponentiated results of binary logistic regression for diagnostic group on HADS/PSS outcome.

	HADS Outcome	PSS Outcome
	Odds Ratio (95% CI)	Odds Ratio (95% CI)
LSD-Other	6.06 (1.64–24.96)	2.85 (0.82–10.37)
RUSP-NBS	3.44 (1.00–12.88)	3.20 (0.96–11.10)
Control	0.49 (0.13–1.93)	0.24 (0.09–1.16)
Northeast	0.32 (0.08–1.14)	0.92 (0.26–3.24)
South	0.53 (0.15–1.77)	2.13 (0.66–7.01)
West	1.48 (0.47–4.64)	1.80 (0.61–5.39)
Less than $34,999	0.37 (0.09–1.44)	0.30 (0.07–1.21)
$50,000–$99,999	1.54 (0.61–3.97)	0.78 (0.34–2.02)
More than $100,000	0.27 (0.05–1.24)	0.93 (0.23–3.80)
All other races	2.94 (0.80–11.37)	
White (Non-Hispanic)	0.62 (0.20–1.90)	
Relationship to the child (mother)		0.66 (0.29–1.46)
		
Major life changes (yes)		0.50 (0.14–1.63)
		
Stay at home parent		0.50 (0.11–1.97)
Unemployed/unable to work		6.52 (0.51–87.55)

## Data Availability

De-identified data used in this analysis can be found in the Supplementary Materials.

## Supplementary Materials

The following supporting information can be downloaded at: https://www.mdpi.com/article/10.3390/ijns8040059/s1, Table S1: Breakdown of Disorders Represented in Study Population; Table S2: De-Identified Study Data.
